# The effectiveness of online educational interventions on impostor syndrome and burnout among medical trainees: a systematic review

**DOI:** 10.1186/s12909-024-06340-y

**Published:** 2024-11-22

**Authors:** Chun-Lun Hsu, Cheng-Heng Liu, Chih-Chung Huang, Huey-Ling Chen, Yen-Lin Chiu, Chih-Wei Yang

**Affiliations:** 1 Center for General Education, National Defense Medical Center, Taipei, Taiwan; 2 Graduate Institute of Medical Sciences, National Defense Medical Center, Taipei, Taiwan; 3 Department of Medical Education & Bioethics, National Taiwan University College of Medicine, No. 1, Section 1, Renai Road, Taipei, Taiwan; 4 Department of Medical Education, National Taiwan University Hospital, Taipei, Taiwan; 5 Department of Emergency Medicine, National Taiwan University Hospital, Taipei, Taiwan; 6 Department of Psychiatry, Tri-Service General Hospital, National Defense Medical Center, Taipei, Taiwan

**Keywords:** Impostor syndrome, Burnout, Medical education, Online interventions, Systematic review

## Abstract

**Background:**

Impostor syndrome and burnout are highly prevalent among medical students and trainees, significantly impacting their mental health and professional development. The advent of online educational interventions provides a promising solution, offering accessibility and flexibility to tackle these issues. This systematic review aims to evaluate the effectiveness of online educational interventions in alleviating impostor syndrome and burnout among medical learners.

**Methods:**

A comprehensive literature search was conducted across PubMed, Cochrane Library, Embase, Scopus and PsycInfo, identifying relevant studies published up to March 2024. Studies focusing on online interventions targeting impostor syndrome and burnout among medical students, residents, and fellows were included, and their quality was assessed using the Medical Education Research Study Quality Instrument (MERSQI).

**Results:**

Among the screened studies, six met our inclusion criteria, comprising four randomized controlled trials, one qualitative study, and one mixed-methods study. Their mean MERSQI score was 14.67 (SD 1.23), indicating a high methodological quality. The interventions adopted in these studies varied, including group coaching sessions, workshops, and provision of educational resources. Notably, two randomized trials demonstrated significant reductions in impostor syndrome symptoms after online interventions, compared with the control groups. On the other hand, results for burnout outcomes were equivocal, with some studies reporting improved emotional exhaustion scores and decreased burnout risk, while others found no significant differences.

**Conclusions:**

Current evidence suggests that structured online educational interventions, particularly those incorporating coaching and cognitive reframing strategies, can effectively reduce impostor syndrome among medical trainees. However, the impact on burnout remains inconclusive. Further research is needed to optimize online program components and implementation strategies to comprehensively address both impostor syndrome and burnout in this population.

**Clinical trial number:**

As this is a systematic review rather than a clinical trial, no clinical trial number is applicable. Nonetheless, this systematic review has been prospectively registered with PROSPERO (registration number: CRD42024541034), in line with best practice recommendations for systematic reviews.

**Supplementary Information:**

The online version contains supplementary material available at 10.1186/s12909-024-06340-y.

## Introduction

Compelling evidence has unveiled the alarming prevalence of impostor syndrome (IS) within the medical profession. This psychological phenomenon, characterized by unrelenting self-doubt and a deep-seated sense in one’s intellectual fraudulence, places a significant burden on medical professionals and students [1, 2]. First identified by Dr. Pauline R. Clance and Suzanne A. Imes in 1978, IS manifests as an inability to internalize accomplishments, leading individuals to attribute their success to external factors such as luck or timing. This pattern often results in a vicious cycle of overcompensation and burnout, especially in fields that demand high achievement, such as medicine.

Within the competitive landscape of the medical community, self-esteem is closely tied to professional success. The prevalence of heroism and peer competition in the medical field can exacerbate sentiments of inadequacy among students and young physicians [3, 4]. Studies have shown that medical students and clinicians experience higher rates of depression, anxiety, and burnout compared to non-medical professions, particularly among female professionals, partly attributed to IS [4, 5]. Comparative studies have further delved into the experiences of IS between physicians and the general population, revealing that physicians are more susceptible to IS, which is closely linked to increased burnout, decreased job satisfaction, and suicidal ideation [1, 6, 7]. These findings call for a reassessment of professional standards towards a more supportive medical education culture that reduces perfectionism [8].

IS should be viewed as a spectrum rather than a binary state, acknowledging its dynamic nature influenced by various external and internal factors. Educators are urged to develop a nuanced understanding of IS, particularly recognizing its gender-specific manifestations [9]. Qualitative research exploring the challenges faced by young trainee doctors such as postgraduate year (PGY) doctors or residents highlights the dilemma between ‘fitting in’ and ‘standing out’ during critical professional transitions. This dilemma substantially contribute to both IS and burnout, emphasizing the necessity for better institutional support for trainees during these transitional periods [3].

Recent medical studies show that IS is common among doctors and medical trainees, significantly impacting their mental health and increasing their risk of burnout. Research indicates that 22–60% of medical trainees and practicing physicians experience IS, which is linked to higher burnout rates. Studies also reveal strong connections between IS, unhealthy perfectionism, and suicidal thoughts in medical students. Importantly, IS appears to bridge the gap between excessive perfectionism and suicidal thinking. These findings highlight how closely IS, burnout, and mental health are intertwined in medical education, emphasizing the need to address these issues in the medical field [9, 10]. The relationship between IS and burnout in medical trainees reveals a complex interplay of psychological and environmental factors. Contributors to IS, such as role ambiguity, inadequate preparation, and challenges during professional transitions, closely parallel and potentially amplify the drivers of burnout, including chronic work-related stress and insufficient support systems [11, 12]. This relationship appears to be bidirectional and self-reinforcing: IS can increase vulnerability to burnout, while experiences of burnout can intensify feelings of IS. As medical trainees strive for success, their ambition may lead to work overload and neglect of self-care, further exacerbating both IS and burnout symptoms. The resulting cycle can significantly impair professional performance, affecting the quality of patient care, diminishing job satisfaction, and potentially leading to attrition from medical careers [13, 14]. Moreover, this cycle can have far-reaching consequences on the overall well-being of healthcare professionals and the broader healthcare system. Recognizing and addressing the combined impact of IS and burnout is crucial for promoting well-being and fostering healthier work environments in medical education and practice.

Although no single intervention has been confirmed to effectively alleviate IS, numerous studies have proposed strategies aimed at mitigating it [15, 16]. These strategies range from documenting one’s accomplishments to engaging in therapy to recognize and change behaviors associated with IS [16–20]. Despite past research summarizing educational measures to improve IS or burnout, there remains a need to further explore the potential of online interventions, given the rise of web-based learning in medical education [21, 22]. However, the impact and optimal format of such online interventions necessitate additional investigation. In the post-pandemic era, the field of medical education has witnessed a surge in online resources, with online courses emerging as an inclusive medium transcending physical boundaries. Online resources, including worksheets and educational materials, provide tools to assist individuals in combating feelings of fraudulence and self-doubt [5]. Hence, the development of accessible online educational interventions is crucial for supporting individuals struggling with these emotions in both their professional and personal lives [23].

Despite the growing body of research on IS and burnout in medical education, a significant gap remains in understanding the effectiveness of online educational interventions in tackling these issues. Although the post-pandemic era has led to a surge in web-based learning resources, there has been little systematic evaluation of their impact on IS and burnout among medical students and trainees. This review aims to address this gap by assessing the available evidence on the efficacy of online courses in alleviating these challenges. Through a systematic review, we seek to gather comprehensive insights to inform the development of tailored, evidence-based online interventions, ultimately improving the wellbeing of current and future medical professionals—a critical need in the evolving landscape of medical education.

## Methods

This study aims to comprehensively assess and synthesize the literature concerning the impact of online educational intervention on IS and burnout among medical learners. A systematic review methodology was employed to enable the methodical identification and evaluation of all relevant evidence that meets predetermined eligibility criteria. This review was conducted following the guidance outlined in the Preferred Reporting Items for Systematic Reviews and Meta-Analyses (PRISMA) statement. This systematic review has been prospectively registered with PROSPERO (ID: CRD42024541034).

### Literature search strategy

A systematic search was conducted across PubMed, Cochrane Library, Embase, Scopus and PsyInfo to identify relevant studies available online as of March 2024. The search strategy incorporated a wide range of terminology related to the target populations (e.g., medical students, residents, fellows), conditions of interest (e.g., impostor phenomenon, IS, burnout), and setting (e.g., undergraduate medical education, graduate medical education). While the PICO framework is commonly employed in systematic reviews, specific comparisons and outcomes were not predefined to ensure a comprehensive and inclusive search. The complete search strategies, including all search terms utilized, can be found in the supplementary appendix.

The review question was formulated as follows: “What is the effectiveness of online educational interventions in reducing IS and burnout among medical learners?” This question guided our systematic review process, focusing on the efficacy of online interventions in addressing IS and burnout among medical trainees. The population of interest for this systematic review comprised medical students, residents and fellows in undergraduate or graduate medical education programs. The interventions under scrutiny were online interventions, such as web-based modules, virtual workshops or seminars, and online coaching and mentoring, aimed at addressing both IS and burnout. Comparisons were drawn between no intervention, face-to-face interventions, or other modalities of interventions, including traditional lectures, self-help materials, and peer support groups. The outcomes of interest included both quantitative and qualitative aspects, examining the effects of online educational interventions on IS and burnout. Quantitative measures included IS symptoms (e.g., self-perceived intellectual phoniness, competence) and burnout indicators (e.g., emotional exhaustion, depersonalization, personal accomplishment). Qualitative outcomes exploring experiences and perceptions of the online interventions were also assessed.

### Eligibility

In order to fulfill the inclusion criteria for our review, studies were required to meet the following criteria:

1. The primary focus of the study was on IS and burnout among medical students and trainees, with an intervention specifically targeting the population of medical students, residents, and fellows.
2. The intervention involved an online component, defined as a structured digital educational program delivered either exclusively online or in a hybrid format combining online and in-person elements. These online components could be delivered through synchronous (real-time/live) or asynchronous (self-paced) formats.

The reported outcomes of the studies were related to:

1. Severity of IS, assessed using validated instruments such as the Young Impostor Syndrome Scale [2].
2. Burnout levels, evaluated through validated metrics including, but not limited to, the Professional Fulfillment Index and Maslach Burnout Inventory [24–26].

The focus of our review was specifically on online program interventions for IS and burnout among medical students and trainees. Articles were excluded if they did not meet the predefined criteria for population, intervention, comparison, and outcome. To ensure a comprehensive review, we included studies published in all languages, using translation services when necessary. Non-peer reviewed literature was excluded to maintain the scientific rigor of our analysis.

### Study selection

Two study authors (C.H.L & C.L.H) independently reviewed all titles and abstracts, selecting relevant studies using Rayyan, a free web and mobile application specifically designed to expedite the initial screening process for systematic reviews [27]. Our study employed Rayyan’s automation tool to efficiently filter out off-topic papers from the systematic review. By establishing exclusion criteria based on specific keywords and study types, and subsequently uploading the dataset, the automation tool was utilized to highlight key elements in titles and abstracts during the initial screening phase. This method incorporates a semi-automation process to assist researchers in filtering searches more efficiently. In instances of disagreement, both authors separately re-evaluated the full text to decide on the eligibility for inclusion. If a consensus could not be reached, a thorough re-assessment and detailed discussion of the text took place, with the final decision aligning with the pre-established inclusion and exclusion criteria. Should the disagreement persist, a third senior author (C.W.Y) was consulted to resolve the issue as an arbitrator.

### Data extraction

The details of included studies were systematically extracted using a standardized form. The extracted information encompassed four key domains: (1) publication details, including the journal name and author; (2) study characteristics, such as the year of publication and country of origin; (3) participant demographics, including number of participants and their level of training; and (4) outcomes, covering major results and key findings.

### Quality appraisal

The methodological quality of all included studies was evaluated using the Medical Education Research Study Quality Instrument (MERSQI) [27, 28], a validated tool designed for assessing studies in the field of medical education. While the MERSQI was originally developed for quantitative research, we adapted its framework to assess qualitative and mixed-methods studies as well. This adaptation allowed us to evaluate all studies consistently, focusing on aspects such as study design, sampling, data collection methods, and analysis rigor across different methodologies. By applying the MERSQI to all studies, we maintained a uniform quality assessment approach while still acknowledging the unique characteristics of each research design.

### Data synthesis

Given the heterogeneity of study designs, interventions, and outcome measures, we conducted a narrative synthesis of the evidence [29, 30]. This approach facilitated the integration of both quantitative and qualitative findings.

We systematically categorized and analyzed the extracted data, identifying key themes and patterns across studies. The synthesis focused on evaluating the effectiveness of online educational interventions in reducing IS and burnout among medical learners. We assessed the strength of evidence, considering the methodological quality of each study.

This narrative approach enabled a comprehensive analysis of the existing literature, elucidating both consistencies and disparities in the findings [31]. The synthesis was structured to provide a clear, evidence-based summary of the current state of knowledge in this field.

## Results

**Fig 1:**
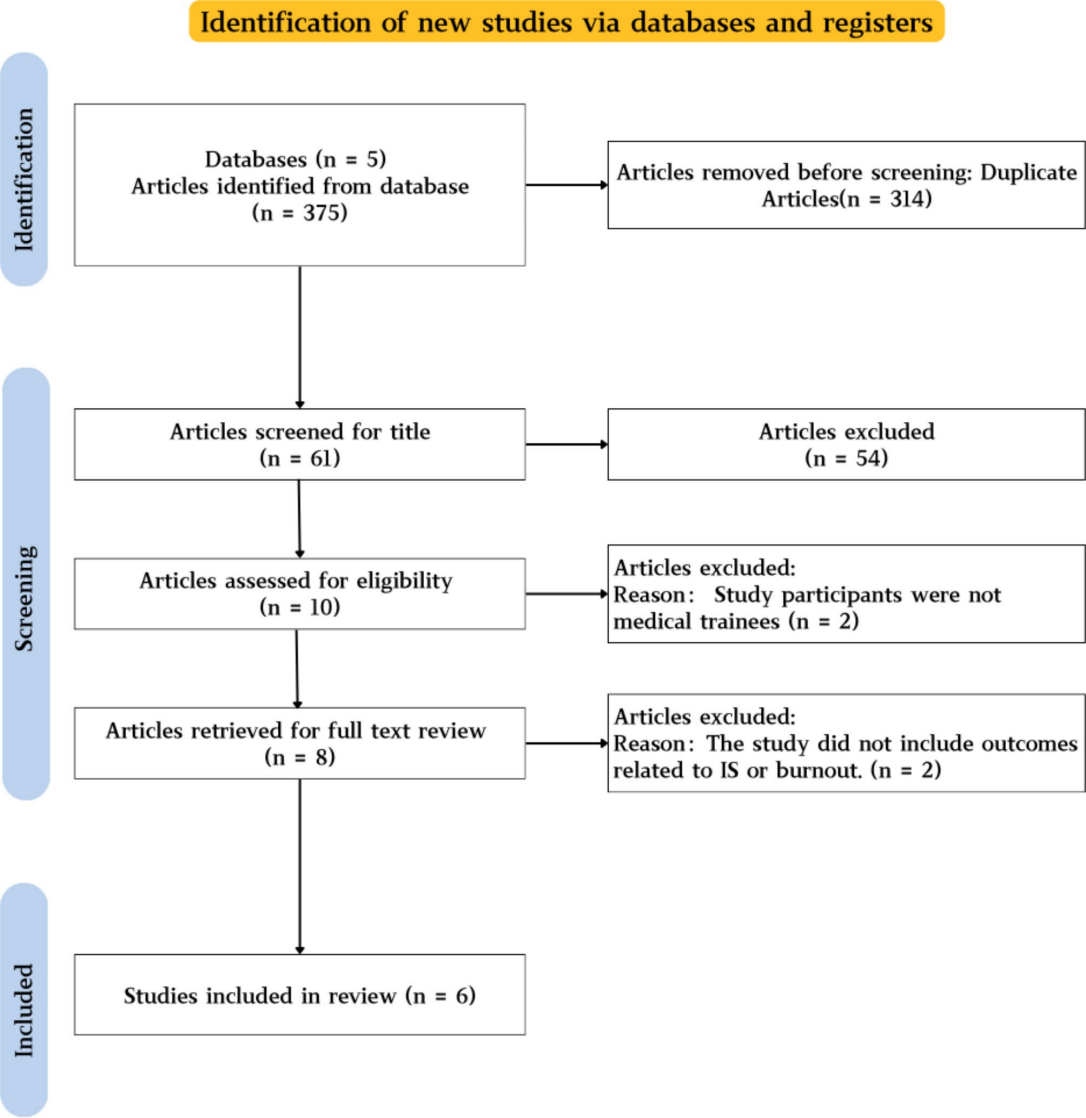
Preferred reporting items for systematic reviews and meta-analyses (PRISMA) flow diagram

The initial literature search identified 375 potentially relevant articles. After screening titles and abstracts, 10 articles were selected for full-text review. Subsequently, 6 studies satisfactorily met all eligibility criteria and were included in the systematic review. The study selection process is illustrated in the PRISMA flow diagram (Fig. 1).

### Overview of included studies

The 6 studies selected for inclusion in this analysis were published between 2018 and 2023. The majority of these studies were published in the last 5 years. This trend indicates a rise in the investigation of online educational interventions for addressing IS and burnout among medical learners over the past decade (Table 1) [32–37].

**Table 1:** Characteristics of studies in a systematic review of Online Educational interventions for Impostor Syndrome and Burnout in Medical students and trainees

Author / Year/ Country	Participants & Methods	Major Outcomes Measurement	Secondary Outcomes Measurement	Intervention	Key Findings
Palamara, Kerri, et al.2023 [32] the U.S.A	237 Female Surgery Residents (Postgraduate Year, PGY)/ Randomized Controlled Trial	Professional Fulfillment and Well-Being	The quartile of the burnout subscale of the Professional Fulfillment Index (PFI).	9-month, 3 coaching sessions	No significant differences in burnout found between intervention and control groups.
Mann, Adrienne, et al.2023 [33] the U.S.A	1,017 Female PGY/ Randomized Clinical Trial	Young Impostor Syndrome scale	Maslach Burnout Inventory (MBI)	a 4-month, web-based group-coaching program (3 to 4 live group coaching calls per week, approximately 5 h per week)	Group coaching intervention led to greater reductions in impostor syndrome scores relative to control group. Qualitative data also supported decreases in impostor syndrome. Group coaching significantly reduced emotional exhaustion and depersonalization subdomain scores of burnout compared to controls. Also found 18% reduction in overall burnout risk.
Fainstad, Tyra, et al.2022 [34] the U.S.A	101 Female PGY/ Randomized Clinical Trial	Young Impostor Syndrome scale	Maslach Burnout Inventory (MBI)	a 6-month, web-based group-coaching program	Online group coaching program significantly reduced symptoms of impostor syndrome compared to control group. Improved emotional exhaustion subdomain of burnout after online group coaching intervention.
Mann, Adrienne, et al.2022 [35] the U.S.A	50 Female PGY/ Qualitative Study		Growing self-compassion Managing imposter syndrome and perfectionism Reduce the overall feeling of burnout	6-month, web-based, group coaching	Qualitative study found coaching helped alleviate anxiety, frustration, feeling overwhelmed and perfectionism.
Danilewitz, Marlon, et al.2018 [36] Canada	52 students who enrolled in the study, 36 (69.2%) were female Most students (79.9%) were in their first (*n* = 21) or second (*n* = 20) year of medical school./ Prospective Pilot Cohort Study		Maslach Burnout Inventory (MBI)	from a minimum of 7weeks to a maximum of 4 months	Providing online wellbeing resources did not improve burnout compared to controls.
Johnson, Judith, et al.2023 [37] the U.K	115 (93/80.9% women) Medical students/ Mixed-methods Study		Oldenburg Burnout Inventory (OLBI)	two, 2-hour online group workshops and a one-to-one coaching call over 4 weeks	Significant pre-post reductions in burnout were observed after an 8-hour wellbeing workshop, sustained over 12 months.

The mean MERSQI score for the included studies was 14.67 (SD 1.23) out of 18 (see Table 2), indicating a high level of methodological quality in the medical education research studies analyzed, despite the diverse range of study designs employed. The MERSQI serves as a validated tool used to assess the methodological quality of medical education research studies. It evaluates studies based on six domains: study design, sampling (including institutions and response rate), type of data, validity of evaluation instrument, data analysis sophistication, and study outcomes.

Among the six analyzed studies, four (Palamara et al., 2023; Mann et al., 2023; Fainstad et al., 2022; and Danilewitz et al., 2018) employed a randomized controlled trial design. Mann et al. (2022) undertook a qualitative study, while Johnson et al. (2023) used a mixed-methods approach. The majority of the studies were conducted within a single institution, with the exception of Mann et al., 2023, which involved 26 institutions. All studies achieved a response rate between 50% and 74%.

In addition, data collection in all studies was performed through participants assessments. Five studies (Palamara et al., 2023; Mann et al., 2023; Fainstad et al., 2022; Danilewitz et al., 2018; and Johnson et al., 2023) demonstrated internal structure validity for the evaluation instrument, while one study (Mann et al., 2022) established content validity. Moreover, most studies employed data analysis techniques beyond descriptive analysis, except for Danilewitz et al. (2018), which solely utilized descriptive analysis. The outcomes measured in all studies included satisfaction, attitudes, perceptions, opinions, general facts, and behaviors.

**Table 2:** Medical Education Research Study Quality Instrument (MERSQI) results

MERSQI Domain	Response Item (Points)	Palamara, Kerri, et al. 2023	Mann, Adrienne, et al. 2023	Fainstad, Tyra, et al. 2022	Mann, Adrienne, et al. 2022	Danilewitz, Marlon, et al. 2018	Johnson, Judith, et al. 2023
Study design	Single group cross-sectional or single group post-test only (1)	-	-	-	-	-	1
	Randomized controlled trial (3)	3	3	3	-	3	-
Sampling: Institutions	1 institution (0.5)	0.5	0.5	0.5	0.5	-	0.5
	3 or more (1.5)	-	-	-	-	1.5	-
Sampling: Response rate	NA (—)	-	-	-	-	-	-
	50–74% (1)	1	1	1	1	1	1
Type of data	Assessment by study participant (1)	1	1	1	1	1	1
	Objective (3)	-	-	-	-	-	-
Validity of evaluation instrument	Internal structure(5)	5	5	5	-	5	5
	Content(5)	-	-	-	5	-	-
	Relationships to other variables(5)	-	-	-	-	-	-
Data analysis: Sophistication	Descriptive analysis (1)	-	-	-	-	1	-
	Beyond descriptive (2)	2	2	2	2	-	2
Outcomes	Satisfactions, attitudes, perceptions, opinions, general facts (1)	1	1	1	1	1	1
	Behaviors (2)	2	2	2	2	2	2
Total		15.5	15.5	12.5	15.5	15.5	13.5

Table 3 presents the integrated MERSQI scores of the six included articles, summarizing the distribution of studies across response item category within the six MERSQI domains. High interrater reliability for quality assessment was observed (ICC = 0.94, 95% CI 0.87–0.97). Additionally, most studies utilized a randomized controlled trial design (*n* = 5, 83.3%), with single institution sampling being the common practice (*n* = 5, 83.3%), while one study (16.7%) opted for multi-site sampling. The majority of studies reported response rates over 50% (*n* = 5, 83.3%). All studies relied solely on self-reported assessments (*n* = 6, 100%), without incorporating objective outcome measures. The validity evidence was predominantly strong, based on internal structure (*n* = 5, 83.3%) or content (*n* = 1, 16.7%). All studies used appropriate data analysis methods (*n* = 6, 100%), though one study exclusively employed descriptive techniques. Sophisticated statistical approaches beyond descriptive analysis were prevalent in most studies (*n* = 5, 83.3%). The evaluation of satisfaction, attitudes, perceptions, and behaviors emerged as the predominant outcomes evaluated in all studies (*n* = 6, 100% each).

**Table 3:** Integrated MERSQI scores of Articles (N, %)

MERSQI Domain	Response Item (Points)	No. of Studies	(%)
Study design	Single group cross-sectional or single group post-test only (1)	1	16.7%
	Randomized controlled trial (3)	5	83.3%
Sampling: Institutions	1 institution (0.5)	5	83.3%
	3 or more (1.5)	1	16.7%
Sampling: Response rate	NA (—)	1	16.7%
	50–74% (1)	5	83.3%
Type of data	Assessment by study participant (1)	6	100.0%
	Objective (3)	0	0.0%
Validity of evaluation instrument	Internal structure (5)	5	83.3%
	Content (5)	1	16.7%
	Relationships to other variables (5)	0	0.0%
Data analysis: Sophistication	Descriptive analysis (1)	1	16.7%
	Beyond descriptive (2)	5	83.3%
Outcomes	Satisfactions, attitudes, perceptions, opinions, general facts (1)	6	100.0%
	Behaviors (2)	6	100.0%

### Narrative synthesis of the studies

This review encompassed six studies examining online educational interventions for medical trainees, predominantly employing randomized controlled trial designs (4 out of 6), with one qualitative and one mixed-methods study. Most were conducted in single institutions, potentially limiting generalizability but allowing for controlled intervention implementation. Response rates ranged from 50 to 74%, with data collection uniformly based on self-reported assessments. The studies demonstrated strong validity, with five establishing internal structure validity and one focusing on content validity. Analysis methods were generally sophisticated, with only one study limited to descriptive statistics. Outcomes measured consistently across studies included satisfaction, attitudes, perceptions, and behaviors, providing a comprehensive view of intervention impacts. The MERSQI scores indicated high interrater reliability and overall high-quality research designs. While the studies showed robustness in experimental approaches and validity measures, the prevalence of single-institution settings and reliance on self-reported outcomes suggest areas for future research expansion. This synthesis reveals a developing standardization in researching online educational interventions for medical trainees, with opportunities to enhance the breadth and depth of findings through multi-institutional studies and incorporation of objective outcome measures.

### Online course

Palamara et al. (2023) and Mann et al. (2023) conducted studies to examine the effectiveness of multi-session individual and group coaching programs. Palamara et al. tested a program consisting of 3 sessions held over a period of 9 months, while Mann et al. evaluated a 4-month program. These coaching programs were overseen by qualified physicians and facilitated by trained physician coaches. Furthermore, Fainstad et al. (2022) utilized a 6-month online group coaching program customized specifically for female residents. In contrast, Johnson et al. (2023) and Danilewitz et al. (2018) evaluated less intensive educational interventions, such as a single 8-hour workshop and the provision of online wellbeing resources, respectively. Finally, Mann et al. (2022) conducted a qualitative analysis of a cohort who participated in the Better Together Physician Coaching program, allowing further exploration of participants’ experiences in the group coaching model.

Across the variety of programs, common elements were identified, which included the promotion of reflection, self-awareness, and self-care skills. Also, these programs focused on building peer community and support, addressing unique challenges faced by women in the field of medicine, and providing tailored content from experienced physician coaches and mentors. The diversity array of interventions and evaluated outcomes significantly enriches our understanding of the efficacy and essential components of coaching and educational initiatives specifically designed for this demographic.

### Impostor syndrome

Two RCTs conducted by Fainstad et al. (2022) and Mann et al. (2023) found that educational interventions yield a substantial reduction in symptoms associated with IS when compared to control groups. Furthermore, qualitative data obtained from Mann et al. (2022) provided additional support for the reduction of IS and perfectionism following the intervention.

### Burnout

The findings regarding burnout outcomes yielded a varied perspective. Palamara et al. (2023) and Danilewitz et al. (2018) found no statistically significant differences in burnout between the intervention and control groups. Conversely, Fainstad et al. (2022) did reported a notable improvement in emotional exhaustion subdomain scores, while Mann et al. (2023) identified an 18% decrease in burnout risk post-intervention. In addition, qualitative insights from Mann et al. (2022) highlighted the efficacy of online coaching in addressing burnout by providing tools to manage emotions such as anxiety, frustration, and feeling overwhelmed. Lastly, Johnson et al. (2023) observed significant reductions in burnout levels pre-and post-intervention, which were sustained over time.

## Discussion

This review summarizes the current evidence concerning online educational interventions targeting IS and burnout among medical trainees. A series of rigorous RCTs have demonstrated that focused training and coaching programs successfully reduce IS symptoms and improve individuals’ mental health [33, 34, 38, 39]. Moreover, qualitative findings have further enriched these results by underscoring participants’ enhanced capacity to reframe maladaptive thoughts and expectations [35]. Together, these findings are consistent with prior literature that emphasizes the effectiveness of cognitive reframing strategies in ameliorating psychological distress and negative self-perception [39–41]. For medical trainees experiencing IS or burnout, this approach involves recognizing and adjusting negative thought patterns.

Previous reviews have identified effective educational interventions for addressing IS, including workshops incorporating self-reflection and group-guided exercises, coaching, and structured supervise [22, 42]. These interventions share common themes of individual coping strategies, peer-to-peer support, and institutional initiatives [22, 43–46]. Furthermore, online interventions offer additional benefits in terms of increased accessibility, flexibility, and scalability compared to traditional in-person workshops [33, 34, 44, 46]. An online course allows participants to attend in a more comfortable and undisturbed environment, reducing interpersonal pressure, which can increase motivation and effectiveness for learning [47]. However, the lack of face-to-face interaction may lead to reduced social presence and diminished opportunities for spontaneous discussions and networking [48]. To address this, online interventions can incorporate interactive elements such as virtual breakout rooms, discussion forums, and scheduled video conferencing sessions to foster a sense of community and maintain human connections [49]. While these digital solutions may not fully replicate in-person interactions, they can help mitigate the loss of interpersonal relationships in the online educational format. The design of these online courses is grounded in cognitive-behavioral principles and social learning theory, which emphasize the role of thoughts, beliefs, and social influences in shaping behavior and emotional well-being [33–35, 37, 46].

Nevertheless, the interventions exhibited varying effects on burnout, with several trials showing no significant differences in burnout levels compared with control groups [33, 34]. In contrast, other studies utilizing coaching models or addressing contributing emotional factors demonstrated more positive effects in reducing burnout [38, 45, 50]. Recent research suggests complex interrelationships between IS and burnout [43, 51, 52]. While targeting IS may alleviate burnout to some extent, it is important to recognize that burnout stems from multiple causes beyond IS, such as high workload, limited autonomy, inadequate support, poor work-life balance, emotional exhaustion, and administrative burdens [50, 53, 54]. Therefore, directly targeting underlying factors like IS may produce more robust effects compared to indirect approaches for mitigating burnout. However, a comprehensive approach addressing various organizational and individual factors may be more effective in mitigating burnout among medical trainees [7, 55]. Further research is needed to deepen our understanding of these factors and their impact on burnout reduction in medical trainees.

There are several limitations to acknowledge in this study. Most educational interventions were tested at mono-centric institutions with modest sample sizes, and few studies followed participants longitudinally to assess the sustainability of benefits throughout trainees’ careers. Hence, there is a necessity for larger, multi-site effectiveness trials in real-world settings. Additionally, a limitation was the predominance of female participants in the majority of studies, likely reflecting the higher prevalence of the IS prevalence women [56]. Recent analyses confirm female gender as a predictor of IS predictor [5, 56–58], underscoring the potential to enhance educational interventions by addressing gender-specific needs. Despite these limitations, controlled research settings have produced promising evidence that structured online training and coaching can positively impact psychological well-being.

In conclusion, this review reveals promising evidence for online programs supporting medical students and trainees in addressing IS and burnout. While the results are encouraging, it’s important to acknowledge the limited number of studies included. This small pool of research reflects the emerging nature of this field and highlights opportunities for growth.

Moving forward, we see potential in developing online interventions that foster self-compassion and mental well-being among medical learners. Further research is needed to refine these programs and understand their long-term impact, particularly in addressing IS and burnout.

As we continue to explore this area, we hope to see an expansion of evidence-based online resources that support future physicians throughout their education and career. By equipping medical trainees with tools to manage psychological distress, we aim to nurture compassionate and resilient healthcare professionals. This growing body of research, though still in its early stages, offers a foundation for future studies to build upon, potentially broadening the scope to capture the full landscape of online interventions in medical education.

## Electronic supplementary material

Below is the link to the electronic supplementary material.

Supplementary Material 1

## Data Availability

The datasets produced and analyzed in the present study are not publicly accessible; however they can be obtained from the corresponding author upon a reasonable request.

## Abbreviations

IS: Impostor syndrome
