# Supplementary material for: The effectiveness of online educational interventions on impostor syndrome and burnout among medical trainees: a systematic review
Source: BMC Med Educ. 2024 Nov 22;24:1349. doi: 10.1186/s12909-024-06340-y (PMC11583500; doi:10.1186/s12909-024-06340-y)
Supplement: Supplementary file 1 — Supplementary Material 1 [file 12909_2024_6340_MOESM1_ESM.docx]

1. PubMed:

((("Impostor Syndrome"[Mesh] OR "impostor syndrome"[tiab] OR "imposter syndrome"[tiab] OR "impostor phenomenon"[tiab] OR "Burnout, Professional"[Mesh] OR burnout[tiab])

AND ("Education, Medical"[Mesh] OR "medical education"[tiab] OR "undergraduate medical education"[tiab] OR "graduate medical education"[tiab] OR "medical student*"[tiab] OR "medical trainee*"[tiab] OR resident*[tiab] OR fellow*[tiab])

AND ("Online Education"[Mesh] OR "online intervention*"[tiab] OR "web-based intervention*"[tiab] OR "e-learning"[tiab] OR "distance learning"[tiab])))

N=14

2. Cochrane Library:

((impostor syndrome OR imposter syndrome OR impostor phenomenon OR burnout)

AND (medical education OR undergraduate medical education OR graduate medical education OR medical student* OR medical trainee* OR resident* OR fellow*)

AND (online intervention* OR web-based intervention* OR e-learning OR distance learning))

N=209

3. Embase:

('impostor syndrome'/exp OR 'impostor syndrome':ti,ab OR 'imposter syndrome':ti,ab OR 'impostor phenomenon':ti,ab OR 'burnout'/exp OR burnout:ti,ab)

AND ('medical education'/exp OR 'medical education':ti,ab OR 'undergraduate medical education':ti,ab OR 'graduate medical education':ti,ab OR 'medical student*':ti,ab OR 'medical trainee*':ti,ab OR resident*:ti,ab OR fellow*:ti,ab)

AND ('online education'/exp OR 'online intervention*':ti,ab OR 'web-based intervention*':ti,ab OR 'e-learning':ti,ab OR 'distance learning':ti,ab)

N=63

4. PsycInfo:

((DE "Impostor Syndrome" OR TI ("impostor syndrome" OR "imposter syndrome" OR "impostor phenomenon") OR AB ("impostor syndrome" OR "imposter syndrome" OR "impostor phenomenon") OR DE "Occupational Stress" OR TI burnout OR AB burnout)

AND (DE "Medical Education" OR TI ("medical education" OR "undergraduate medical education" OR "graduate medical education") OR AB ("medical education" OR "undergraduate medical education" OR "graduate medical education") OR TI ("medical student*" OR "medical trainee*" OR resident* OR fellow*) OR AB ("medical student*" OR "medical trainee*" OR resident* OR fellow*))

AND (DE "Online Education" OR TI ("online intervention*" OR "web-based intervention*" OR "e-learning" OR "distance learning") OR AB ("online intervention*" OR "web-based intervention*" OR "e-learning" OR "distance learning")))

N=5

5. Scopus:

TITLE-ABS-KEY("impostor syndrome" OR "imposter syndrome" OR "impostor phenomenon" OR burnout)

AND TITLE-ABS-KEY("medical education" OR "undergraduate medical education" OR "graduate medical education" OR "medical student*" OR "medical trainee*" OR resident* OR fellow*)

AND TITLE-ABS-KEY("online intervention*" OR "web-based intervention*" OR "e-learning" OR "distance learning")

N=84
